# Fault Diagnosis for Rolling Bearing of Combine Harvester Based on Composite-Scale-Variable Dispersion Entropy and Self-Optimization Variational Mode Decomposition Algorithm

**DOI:** 10.3390/e25081111

**Published:** 2023-07-25

**Authors:** Wei Jiang, Yahui Shan, Xiaoming Xue, Jianpeng Ma, Zhong Chen, Nan Zhang

**Affiliations:** 1 Jiangsu Key Laboratory of Advanced Manufacturing Technology, Huaiyin Institute of Technology, Huai’an 223003, China; 2 Wuhan Second Ship Design and Research Institute, Wuhan 430064, China; 3 Aero Engine Corporation of China, Harbin Bearing Co., Ltd., Harbin 150500, China; mjp930116@163.com

**Keywords:** fault diagnosis, dispersion entropy, VMD, rolling bearing, combine harvester

## Abstract

Because of the influence of harsh and variable working environments, the vibration signals of rolling bearings for combine harvesters usually show obvious characteristics of strong non-stationarity and nonlinearity. Accomplishing accurate fault diagnosis using these signals for rolling bearings is a challenging subject. In this paper, a novel fault diagnosis method based on composite-scale-variable dispersion entropy (CSvDE) and self-optimization variational mode decomposition (SoVMD) is proposed, systematically combining the nonstationary signal analysis approach and machine learning technology. Firstly, an improved SoVMD algorithm is developed to realize adaptive parameter optimization and to further extract multiscale frequency components from original signals. Subsequently, a CSvDE-based feature learning model is established to generate the multiscale fault feature space (MsFFS) of frequency components for the improvement of fault feature learning ability. Finally, the generated MsFFS can serve as the inputs of the Softmax classifier for fault category identification. Extensive experiments on the vibration datasets collected from rolling bearings of combine harvesters are conducted, and the experimental results demonstrate the more superior and robust fault diagnosis performance of the proposed method compared to other existing approaches.

## 1. Introduction

As a widely used agricultural machinery, the combine harvester plays an essential role in the automatic process of crop harvesting [1]. The rolling bearing is a fundamental and an important load-carrying component in the combine harvester and has a significant influence on the stable and reliable operation of equipment [2]. Considering the harsh and variable operating environment, several different types of faults will gradually occur in key parts of the bearing and may lead to serious security incidents if they cannot be treated in a timely manner [3,4]. Therefore, accurate bearing fault diagnosis is of great importance to ensure a continuous and healthy operation state of a combine harvester and has gained much more attention for its significant value of research.

Recently, with the development of machine learning technology, various fault detection and diagnosis approaches have been proposed and have achieved successful applications in engineering practice. In Reference [5], a decentralized SVDD-based fault diagnosis method was presented and the experimental results demonstrated the feasibility of the method. To facilitate the detection of incipient faults, Zhao designed an auxiliary input signal for active fault diagnosis [6]. Generally, the vibration signals of the rolling bearing contain a large amount of information that reflects the actual health states and usually serve as the data inputs of the fault diagnosis model [7]. However, because of the influence of complex working conditions and the system dynamic response, the collected vibration signals present significant characteristics of strong non-stationarity and nonlinearity in most cases [8]. For this reason, various advanced time–frequency signal analysis methods have been applied successfully for signal processing to reduce the complexity of the original signal and further learn useful state information for diagnosis [9]. On this foundation, the redundant information contained in raw signals can be effectively filtered and the corresponding results of signal analysis provide solid support for subsequent feature extraction. The most frequently used time–frequency analysis approaches include the wavelet transform (WT), empirical mode decomposition (EMD), and a set of related improved methods, such as the empirical wavelet transform (EWT) and ensemble EMD (EEMD) [10,11,12,13]. Liang used a WT-based method to extract the fault features and realize the fault diagnosis of a bearing [10]. In Reference [11], an improved EWT approach was presented for bearing diagnosis. Although WT and its improved methods have been widely applied in the fields of diagnosis, the problems of insufficient adaptability and wavelet-based selection still cause some restrictions in practical application. Unlike WT, EMD and its optimized methods have been developed and have effectively achieved adaptive performance [12,13]. Nevertheless, a problem not to be ignored is that the mode mixing phenomenon that exists in the results of signal analysis may cause an obvious decrease in final diagnosis accuracy. Fortunately, as an adaptive algorithm, VMD helps to extract a series of frequency components from the original signal and, at the same time, avoid the influence of mode mixing [14]. The algorithm can determine the relevant bands adaptively and perfectly balance errors between different frequency components to obtain the separation of components from original signals. Zhou proposed a VMD-based method for bearing fault diagnosis and achieved better performance than existing diagnosis technology [15]. In addition, the effectiveness of VMD has also been strongly validated and many successful applications in practical problems can be seen in References [16,17]. Nevertheless, it is worth noting that two important parameters of the VMD algorithm including the number of components and the penalty factor are usually set randomly, which will have a significant influence on the final decomposition results [18]. For this reason, adaptively optimizing these parameters of VMD has become a hot topic in the fields of non-stationary signal processing and fault diagnosis.

Based on the preliminary results of signal analysis, the state features of the bearing can be further captured for diagnosis [19,20,21,22]. Zhao proposed a bearing multi-fault diagnosis method guided by the instantaneous fault characteristic frequency extraction and enhanced instantaneous rotational frequency matching, and the related experiment results validated the effectiveness of the method [19]. Wang developed a bilayer convolutional transfer learning neural network with better generalization performance to effectively extract the fault features [20]. In Reference [21], the bearing fault type was determined directly using the fault characteristic frequency and rotational frequency harmonics. Recently, due to the development of artificial intelligence technology and system dynamics theory, entropy-based feature extraction approaches have become a hot topic to be explored gradually [23,24]. As a transcendental and important statistical concept in many disciplines, entropy can effectively measure the dynamic changes of vibration signal when a fault occurs without the linear hypothesis. Because of the significant advantages, different types of entropies have been developed to automatically capture the effective features from raw signals for fault diagnosis [25,26]. For instance, approximate entropy (AE) was proposed to describe the underlying deterministic changes and further measure the dynamic changes in original signals [27]. Based on this, an AE-based model was proposed to identify the spall-like fault [28]. Limited by the theoretical basis of AE, the quality of the obtained features can be easily affected by the signal length and more similarity would be generated so that the diagnosis cannot give satisfactory results [25]. As the improvements of AE, sample entropy (SE) [29] and permutation entropy (PE) [30] were developed and effectively overcame the limitations of AE. Gao proposed an SE-based method to complete the task of early fault diagnosis of bearings [31]. In Reference [32], a bearing fault feature space was constructed using PE theory. However, some inherent defects for these entropies, such as the problems of boundary discontinuity and amplitude information loss, would also have a significant impact on the final diagnosis results [33,34]. Unlike the above-mentioned entropies, dispersion entropy (DE) has an excellent ability for measuring the irregularity of signals and solves the problems existing in these entropies [35]. Due to the ideal robustness and computation efficiency, DE has been widely applied in the fields of fault diagnosis [36,37,38]. But it should be noted that these different types of entropies are all single-scale analysis methods, without taking the dynamic characteristics in multiple scales into consideration [39]. Specifically, some essential state information may be contained in these features. Considering the advantages of DE, it is a valuable subject where a novel multi-scale feature extraction method integrated with DE should be provided to achieve the goal of accurate fault diagnosis.

**Figure 1:**
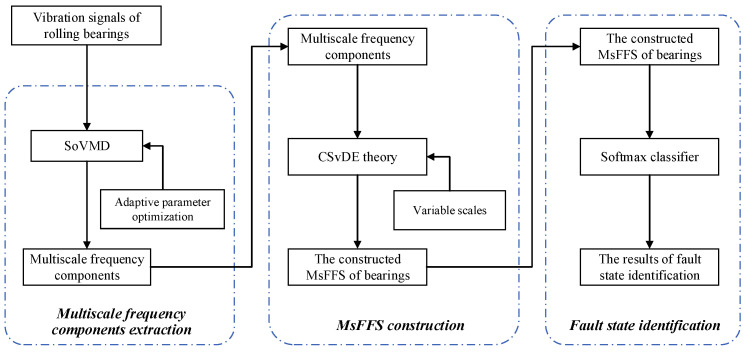
The block diagram of the proposed fault diagnosis method.

The main contribution of this work is the development of a novel rolling bearing fault diagnosis method for a combine harvester based on composite-scale-variable dispersion entropy (CSvDE) and self-optimization VMD (SoVMD) algorithms, systematically blending the nonstationary signal analysis technique and machine learning technology. The block diagram of the proposed method is depicted in Figure 1. In general, the implementation of the proposed method can be divided into three stages, including the first-stage multiscale frequency component extraction, the second-stage MsFFS construction, and the third-stage fault state identification. And on the subject of detail, to eliminate the influence of the strong non-stationarity and nonlinearity of original signals on the diagnosis results, an improved SoVMD algorithm is developed to decompose the original vibration signal into several multiscale frequency components first. It can be seen as a remarkable improvement on the basis of the traditional VMD described in References [14,15,16] so that the parameters of VMD can be adaptively optimized by the SoVMD method. Subsequently, a new CSvDE-based model is designed to construct the multiscale fault feature space (MsFFS) of frequency components. Compared with other entropy-based feature extraction approaches introduced in References [33,34,35], the constructed MsFFS effectively integrates the advantage of variable scales of CSvDE and has great potential to reveal essential information of different fault states. Finally, based on the MsFFS, the softmax classifier is used to identify the fault states of bearings. Overall, the multiscale frequency component extraction can be regarded as the preprocessing stage of the subsequent MsFFS construction, and the acquired MsFFS of bearings should serve as the inputs of the softmax model. Extensive experiments on the vibration datasets collected from rolling bearings of the combine harvester are implemented and the experimental results demonstrate the more superior and robust fault diagnosis performance of the proposed method compared to other existing approaches.

The rest of the work can be briefly introduced as follows. Section 2 describes the research methodology and the general procedure of the proposed method. In Section 3, the experimental results and the corresponding discussion are presented in detail. At the end of the paper, the conclusions are given in Section 4.

## 2. Research Methodology

### 2.1. Self-Optimization VMD Algorithm

#### 2.1.1. VMD Algorithm

VMD is an adaptive non-stationary signal processing method [14]. The *k*-th mode component using VMD can be defined as

(1) $$c_{k}\left(t\right)=A_{k}\left(t\right)\cos\left(\theta_{k}\left(t\right)\right),k=1,2,\cdots ,K$$

where $A_{k}\left(t\right)$ and $\theta_{k}\left(t\right)$ are the instantaneous amplitude and phase, respectively, and K represents the number of mode components. In order to estimate the optimal bandwidth of mode $c_{k}\left(t\right)$, a constrained variational model can be established as [14]

(2) $$\underset{\left\{c_{k},\phi_{k}\right\}}{\min}\left\{\sum_{k=1}^{K}{\left\|\partial_{t}\left[\left(\delta \left(t\right)+\frac{j}{\pi t}\right)*c_{k}\left(t\right)\right]e^{-j\phi_{k}t}\right\|}_{2}^{2}\right\},\;s.t.\;\sum_{k=1}^{K}c_{k}\left(t\right)=s\left(t\right)$$

where $\phi_{k}$ is the center frequency, $\partial_{t}$ is the gradient operation, $\delta \left(t\right)$ is the Dirac function, ∗ represents the convolution operator, j is the imaginary unit, and $s\left(t\right)$ is the raw signal.

To obtain the solution of Equation (2), a penalty factor α and Lagrangian multiplier λ should be introduced as

(3) $$L\left(\left\{c_{k}\right\},\left\{\phi_{k}\right\},\lambda \right)=\alpha \sum_{k=1}^{K}{\left\|\partial_{t}\left[\left(\delta \left(t\right)+\frac{j}{\pi t}\right)*c_{k}\left(t\right)\right]e^{-j\phi_{k}t}\right\|}_{2}^{2}+{\left\|s\left(t\right)-\sum_{k=1}^{K}c_{k}\left(t\right)\right\|}_{2}^{2}+\langle \lambda ,s\left(t\right)-\sum_{k=1}^{K}c_{k}\left(t\right)\rangle$$

Then, the alternate direction multiplier algorithm is considered to solve Equation (3) and the mode $c_{k}\left(t\right)$ can be acquired as

(4) $${\hat{c}}_{k}^{n+1}=\frac{\hat{s}\left(\phi \right)-\sum_{i\neq k}{\hat{c}}_{i}\left(\phi \right)+\frac{\hat{\lambda }\left(\phi \right)}{2}}{1+2\alpha {\left(\phi -\phi_{k}\right)}^{2}}$$

(5) $$\phi_{k}^{n+1}=\frac{\int_{0}^{\infty }\phi {\left|{\hat{c}}_{k}^{n+1}\left(\phi \right)\right|}^{2}d\phi }{\int_{0}^{\infty }{\left|{\hat{c}}_{k}^{n+1}\left(\phi \right)\right|}^{2}d\phi }$$

where n is the number of iterations, and ${\hat{c}}_{k}^{n+1}$, $\hat{s}\left(\phi \right)$, ${\hat{c}}_{i}\left(\phi \right)$, and $\hat{\lambda }\left(\phi \right)$ are the Fourier transforms of $c_{k}^{n+1}\left(t\right)$, $s\left(t\right)$, $c_{i}\left(t\right)$, and $\lambda \left(t\right)$, respectively.

#### 2.1.2. The Developed SoVMD Algorithm

In order to eliminate the influence of strong vibration signal nonlinearity on the diagnosis result, a self-optimization VMD (SoVMD) algorithm is designed to extract the multiscale frequency components from the original signal without the problem of mode mixing and further contributes to learning the inherent characteristics of bearing fault patterns from different scales. More specifically, from the perspective of parameter optimization, the developed SoVMD adopts a hierarchical search structure with adjustable step sizes and effectively achieves the goal of adaptive parameter search (including the number of components K and the penalty factor α), which is a significant improvement compared to the traditional VMD method. The detailed steps of SoVMD can be summarized as follows.

Step 1: Initialize the parameters of the VMD method, including the frequency component $\left\{c^{1}\right\}$, the center frequency $\left\{\phi^{1}\right\}$, and the Lagrangian multiplier $\left\{\lambda^{1}\right\}$, and the search intervals of K and α should be pre-determined.

Step 2: Initialize the parameters related to the searching process, i.e., the maximum number of iterations M, the population size of searching particles P, the initial step size $SS_{0}$, and the initial searching location $\left(X_{0},Y_{0}\right)$.

Step 3: The location $\left(X_{j},Y_{j}\right)$ of the *j*-th particle can be adjusted with a random direction as

(6) $$X_{j}=X_{0}+SS_{i},\;Y_{j}=Y_{0}+SS_{i}$$

with

(7) $$SS_{i}=SS_{0}{\left(\frac{1}{100}\right)}^{\frac{i-1}{M}}$$

where i represents the current iteration number, 1≤i≤M, and $SS_{i}$ represents the step size of the *i*-th iteration. The construction of Equation (7) realizes the dynamic optimization of search step size and further improves the efficiency and accuracy of parameter searching.

Step 4: The distance between the particle location and the coordinate origin $\left(0,\;0\right)$ can be obtained as

(8) $$H_{j}={\left(X_{j}^{2}+Y_{j}^{2}\right)}^{\frac{1}{2}}$$

Step 5: Calculate the concentration value of each particle $Cv_{j}$ using the concentration judgment function, i.e., the fitness function, as

(9) $$Cv_{j}=f_{fitness}\left(\frac{1}{H_{j}}\right)$$

(10) $$f_{fitness}=\frac{1}{R}$$

where R represents the root-mean-square error of the training samples.

Step 6: Based on the search process among the whole population, the optimal concentration value $Cv_{op}$ and the optimal particle location $\left(X_{op},Y_{op}\right)$ are acquired and updated as

(11) $$\left\{\begin{array}{l} Cv=Cv_{op}\\ X_{0}=X_{op}\\ Y_{0}=Y_{op} \end{array}\right.$$

Step 7: Steps 2–7 should be repeated with i=i+1 until the decision condition i=M is met.

Step 8: Through the steps above mentioned, the optimal values of K and α can be obtained. Similar to the implementation process of the VMD method depicted in Reference [14], the raw signal $s\left(t\right)$ can be decomposed into K components with different frequency scales:

(12) $$s\left(t\right)=\sum_{i=1}^{K}c_{i}\left(t\right)$$

**Figure 2:**
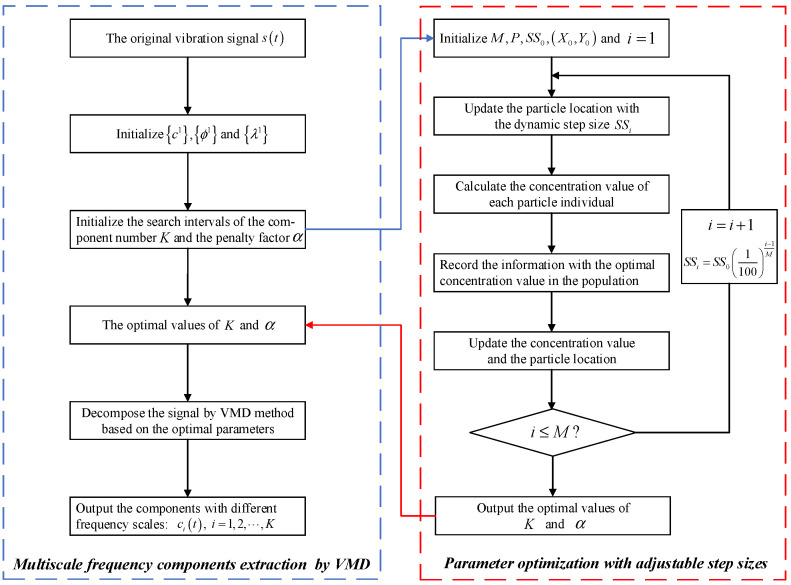
The flowchart of the proposed SoVMD algorithm.

It is worth noting that the whole process of parameter optimization in the proposed SoVMD method can be primarily divided into two stages, i.e., the initial stage with the larger search step sizes and the latter stage with the smaller sizes. Specifically, the large size contributes to accelerate the convergence and strength of the global optimization performance, and the small size can be considered for the purpose of accurate local search. Consequently, because of the dynamic adjustable step size adopting in SoVMD approach, the divergence between the global and local optimization can be effectively balanced and the efficiency and accuracy of parameter optimization can be significantly improved. The flowchart of the proposed SoVMD algorithm can be depicted in Figure 2.

### 2.2. Composite-Scale-Variable Dispersion Entropy

#### 2.2.1. Dispersion Entropy

As a non-linear characteristic indicator, dispersion entropy (DE) can be used to evaluate the irregularity and uncertainty of the signal sequence quantitatively [35]. Based on the advantages of low computation consumption and taking the amplitude’s order and relationship with the theoretical system into consideration, DE helps to obtain more reliable and robust diagnosis results. Given a signal sequence $X\left(t\right)=\left\{x\left(1\right),x\left(2\right),\cdots ,x\left(N\right)\right\}$ (N is the length of the sequence), the DE of this sequence can be calculated by the following steps.

(1) For the original sequence $X\left(t\right)$, a corresponding mapped sequence $U\left(t\right)=\left\{u\left(1\right),u\left(2\right),\cdots ,u\left(j\right),\cdots ,u\left(N\right)\right\}$ can be constructed based on the following formula:

(13) $$u\left(j\right)=\frac{1}{\sqrt{2\pi }\sigma }\int_{-\infty }^{x\left(j\right)}e^{\frac{-{\left(\tau -\mu \right)}^{2}}{2\sigma^{2}}}d\tau$$

where μ is the expectation and σ is the standard deviation. Specifically, the value of $u\left(j\right)$ is between 0 and 1.

(2) Then, for the element $u\left(j\right)$, an integer $v^{m}\left(j\right)$ between 1 and m can be obtained by a linear model:

(14) $$v^{m}\left(j\right)=round\left(m.u\left(j\right)+0.5\right)$$

where m represents the number of categories and $round\left(\cdot \right)$ represents the integer function.

(3) Based on the above equation, an embedding sequence $v^{\gamma ,m}\left(j\right)$ can be defined as follows:

(15) $$v^{\gamma ,m}\left(j\right)=\left(v^{m}\left(j\right),v^{m}\left(j+\xi \right),\cdots ,v^{m}\left(j+\left(\gamma -1\right)\xi \right)\right)$$

where γ is the embedding dimension and ξ is the time delay. Especially, each element of $v^{\gamma ,m}\left(j\right)$ can be mapped into a dispersion mode $\Pi_{\varphi_{0}\varphi_{1}\cdots \varphi_{\gamma -1}}$, in which

(16) $$v^{m}\left(j\right)=\varphi_{0},\;v^{m}\left(j+\xi \right)=\varphi_{1},\;\cdots \;,\;v^{m}\left(j+\left(\gamma -1\right)\xi \right)=\varphi_{\gamma -1}$$

(4) Calculate the relative frequency of each mode by the following formula:

(17) $$f\left(\Pi_{\varphi_{0}\varphi_{1}\cdots \varphi_{\gamma -1}}\right)=\frac{Number\left\{j\left|j\leq N-\left(\gamma -1\right)\xi ,\;v^{\gamma ,m}\left(j\right)\;\mathrm{has}\;\mathrm{type}\;\Pi_{\varphi_{0}\varphi_{1}\cdots \varphi_{\gamma -1}}\right.\right\}}{N-\left(\gamma -1\right)\xi }$$

(5) Based on the definition of Shannon entropy, the DE of sequence $X\left(t\right)$ can be calculated as

(18) $$DE\left(X\left(t\right),\gamma ,m,\xi \right)=-\sum_{\Pi =1}^{m^{\gamma }}f\left(\Pi_{\varphi_{0}\varphi_{1}\cdots \varphi_{\gamma -1}}\right)\ln\left(f\left(\Pi_{\varphi_{0}\varphi_{1}\cdots \varphi_{\gamma -1}}\right)\right)$$

#### 2.2.2. The Proposed CSvDE Theory

Based on the extracted multiscale frequency components, the valuable and inherent features should be learned from these components for accurate fault diagnosis. Considering the complexity of failure causes and the diversity of frequency scales, a composite-scale-variable dispersion entropy (CSvDE) theory is developed and further serves as the effective features for bearing fault diagnosis. Compared with the classical DE approach, the proposed CSvDE method contributes to revealing sensitive characteristics of components from different scales and, at the same time, retains all important information that is essential for accurate diagnosis. The computation principle of CSvDE can be illustrated in detail as follows.

Suppose $c_{i}\left(t\right)=\left\{c_{i}\left(1\right),c_{i}\left(2\right),\cdots ,c_{i}\left(N\right)\right\}$ represents the obtained *i*-th frequency component and *N* represents the length of a component. Some coarse graining sequences can be generated as follows:

(19) $$c_{w,i}^{\zeta }=\frac{1}{\zeta }\sum_{w+\left(i-1\right)\zeta }^{w+\zeta i-1}c_{j},\;1\leq i\leq \frac{N}{\zeta },\;1\leq w\leq \zeta$$

where ζ is the scale factor and w is the order number of coarse graining sequences. To sum up, the CSvDE of component $c_{i}\left(t\right)$ can be finally calculated by the following formula:

(20) $$CSvDE\left(c_{i}\left(t\right),\gamma ,m,\xi ,\zeta \right)=-\sum_{\Pi =1}^{m^{\gamma }}\bar{f}\left(\Pi_{\varphi_{0}\varphi_{1}\cdots \varphi_{\gamma -1}}\right)\ln\left(\bar{f}\left(\Pi_{\varphi_{0}\varphi_{1}\cdots \varphi_{\gamma -1}}\right)\right)$$

where $\bar{f}\left(\Pi_{\varphi_{0}\varphi_{1}\cdots \varphi_{\gamma -1}}\right)=\frac{1}{\zeta }\sum_{w=1}^{\zeta }f_{w}^{\left(\zeta \right)}$ is the average relative frequency of mode Π in the sequence $c_{w}^{\left(\zeta \right)}$.

### 2.3. The Implementation of the Proposed Fault Diagnosis Method

In this paper, a novel fault diagnosis method is proposed for the rolling bearing of a combine harvester. Based on some improved models, including SoVMD and CSvDE, a multiscale fault feature space (MsFFS) can be constructed and the purpose of accurate fault diagnosis can be further achieved. This section illustrates the strategy of MsFFS construction and the detailed implementation procedure of the proposed fault diagnosis method.

#### 2.3.1. The Construction of Multiscale Fault Features Space

Considering the high similarity and complexity between different types of bearing fault signals, it is difficult to obtain accurate diagnosis results only depending on the CsvDE values of raw signal with a single scale factor. For this, a high-dimensional feature pool, written as the MsFFS, can be innovatively constructed to characterize the essential information of fault categories from the perspective of various scales. To give more detail, based on the obtained multiscale frequency components using the SoVMD algorithm, the CSvDE features of these components under different scale factors need to be acquired so that the MsFFS of signal samples can be further established. The generated MsFFS can be denoted as follows, which systematically couples different scales from the levels of the frequency component and dispersion entropy:

(21) $$MsFFS=\left[\begin{array}{l} Sample_{1}\left(CSvDE\left(c_{i},\gamma ,m,\xi ,\zeta_{j}\right)\right);\\ Sample_{2}\left(CSvDE\left(c_{i},\gamma ,m,\xi ,\zeta_{j}\right)\right);\\ \vdots \\ Sample_{S}\left(CSvDE\left(c_{i},\gamma ,m,\xi ,\zeta_{j}\right)\right); \end{array}\right],\;1\leq i\leq K,\;1\leq j\leq L$$

where S and K are the number of samples and frequency components, respectively, and L is the maximum of factor ζ. It can be found from Equation (21) that the dimensionality of the constructed MsFFS is $S\times \left(KL\right)$.

In addition, from Equation (21), it can be seen that there are four parameters that need to be considered in CSvDE and MsFFS, including the embedding dimension γ, the number of category m, the time delay ξ, and the scale factor ζ. Meanwhile, comparing Equations (18) and (21), we can observe that there are three common parameters in DE and CSvDE, i.e., γ, m, and ξ. Based on the principle of DE described in Reference [35], the appropriate value of embedding dimension γ is of great significance to sensitively identify the dynamic changes in the original signal. In other words, too small a γ increases the difficulties for the identification of signal dynamic changes inevitably, while too large a γ easily results in a sluggish response to minor changes. Moreover, the value of category number m should be larger than 1 to guarantee enough dispersion modes in the MsFFS. When it is too small, two amplitude values that are far from each other may be classified into a similar category. But when it is too large, a very small difference may change their category, and the results of MsFFS are easily affected by noise. Also, if γ or m is too large, the computation time is very high. Thus, it is recommended to choose m from 4 to 9 [35,36]. It should be noted that the number of potential dispersion modes $m^{\gamma }$ needs to satisfy the following condition: $m^{\gamma }\leq N$ (N is the length of frequency component $c_{i}$) [35,36]. For the time delay ξ, it is suggested that the corresponding value should be set as 1 [35]. If ξ>1, some important frequency information may be discarded. Consequently, referring to the literature [35,36], these three parameters can be set in this study as γ=4, m=6, and ξ=1. And for the important parameter ζ in the theoretical framework of CSvDE, too small a value is not sufficient to capture essential differences between different types of fault samples and too large a value significantly increases the computation cost. A further analysis of the influence of scale factor ζ on the diagnosis results is shown later in the next section. 

#### 2.3.2. The Procedure of the Proposed Method

**Figure 3:**
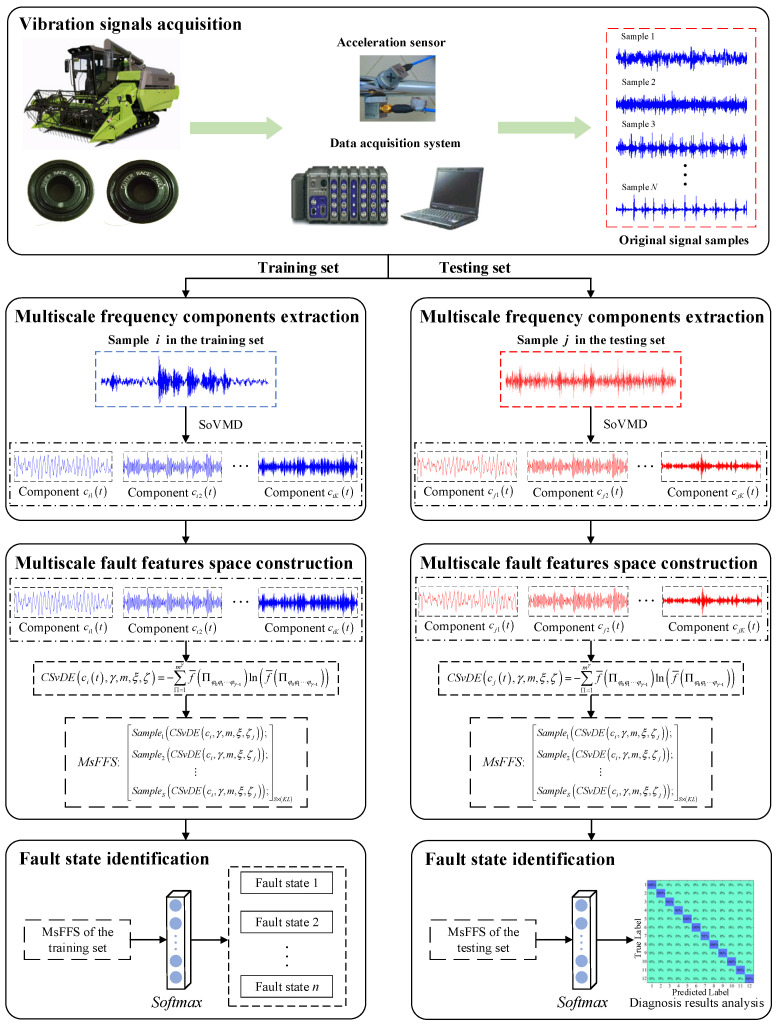
The flowchart of the proposed fault diagnosis method for rolling bearing of combine harvester.

The flowchart of the proposed fault diagnosis method for rolling bearings of combine harvesters is presented in Figure 3. More specifically, due to the strong parameter self-optimization ability and superior decomposition performance of the SoVMD described in Section 2.1, it can be used to decompose the fault signals, and then the MsFFS of bearings is constructed based on the developed CSvDE theory. The implementation procedure of the proposed method is summarized as follows.

(1) The vibration signals of harvester rolling bearings are collected by relevant sensors and the data acquisition system.
(2) The collected vibration signals need to be classified into two parts at random, including the training set and testing set.
(3) Each signal sample in the training set should be decomposed into several multiscale frequency components using the SoVMD algorithm.
(4) On the basis of the analysis of the pre-research experiment, the appropriate value range of scale factor ζ can be determined. Furthermore, in order to guarantee the effectiveness and completeness of the extracted fault information, the CSvDE values of frequency components under different scale factors should be calculated for the construction of MsFFS.
(5) The Softmax classifier is employed to identify the fault category of rolling bearings. For this, the cross-entropy function is considered to calculate the corresponding fault identification loss as [40,41].

(22) $$Loss=-\frac{1}{n}\sum_{i=1}^{n}l_{i}\log\left({\tilde{l}}_{i}\right)=-\frac{1}{n}\sum_{x_{i}\in \left\{x,l\right\}}\sum_{c=1}^{C}P_{x_{i}\mapsto c}\log\left(G_{l}\left(MsFFs\left(x_{i}\right)\right)\right)$$

where $x_{i}$ represents the raw signal sample, $l_{i}$ represents the corresponding truth label, ${\tilde{l}}_{i}$ represents the predicted label of the softmax model, n is the number of samples, C is the number of fault categories, $P_{x_{i}\mapsto c}$ represents the corresponding probability of sample $x_{i}$ belonging to fault category c, and $G_{l}$ represents the softmax classifier.

(6) A testing set is utilized to validate the feasibility and superiority of the proposed diagnosis method.

Through the above diagnosis steps, the specific fault state of a harvester rolling bearing can thus be identified and determined. In essence, the fault diagnosis results provided by the proposed method are equivalent to the classification results of the softmax classifier. In addition, based on the diagnosis results, two commonly used evaluation metrics are calculated to analyze the performance of diagnosis method, including diagnosis accuracy and false alarm rate (FPR) [42,43]. Based on the machine learning theory related to the classification problem, the definitions of these two metrics are presented as follows.

(23) $$Accuracy(\%)=\frac{1}{C}\sum_{i=1}^{C}\left(\frac{TP_{i}+TN_{i}}{M}\times 100\%\right)$$

(24) $$FPR\left(\%\right)=\frac{1}{C}\sum_{i=1}^{C}\left(\frac{FP_{i}}{FP_{i}+TN_{i}}\times 100\%\right)$$

where C is the number of fault categories; $TP_{i}$, $TN_{i}$, and $FP_{i}$ represent the true positives, true negatives, and false positives for the *i*-th fault category, respectively; and M is the total number of testing samples. The larger accuracy and the smaller FPR represent the better performance of the diagnosis method.

## 3. Experiment Validation and Results Discussion

### 3.1. Dataset Description

**Figure 4:**
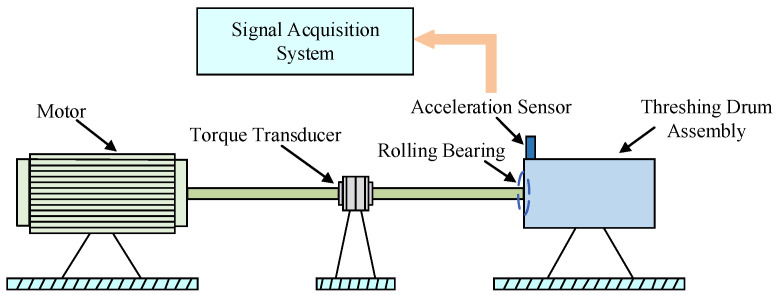
The structure diagram of experimental platform.

**Figure 5:**
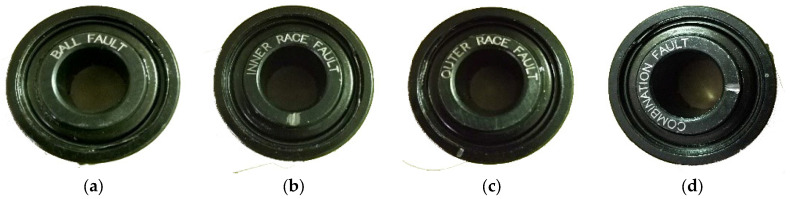
Different types of rolling bearing faults: (**a**) roller fault (RF); (**b**) inner ring fault (IRF); (**c**) outer ring fault (ORF); (**d**) combination fault (CF).

**Figure 6:**
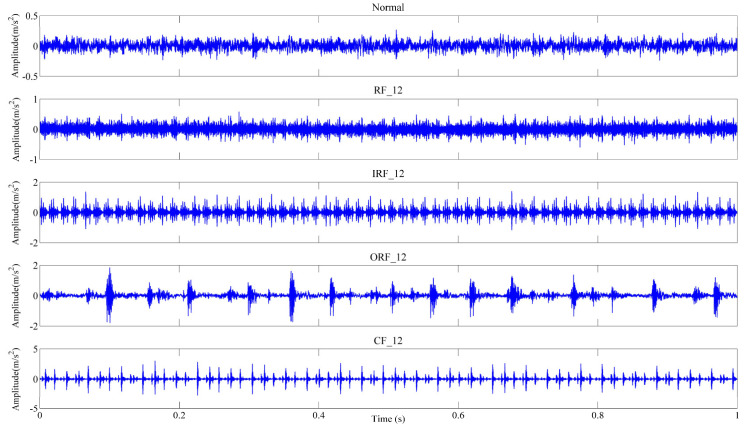
The time-domain waveforms of raw vibration signals for five states.

In this study, the vibration signals collected from rolling bearings that are installed on the threshing drum assembly of the combine harvester can be utilized for experimental analysis. The structure diagram of the test platform is depicted in Figure 4, which mainly consists of a motor, a torque transducer, a drum assembly, and a signal acquisition system. As shown in Figure 4, the acceleration sensor is attached to the vertical direction at the front end of the drum assembly and utilized to acquire the vibration signals of rolling bearings under different operation conditions with a sampling frequency of 10 kHz. Specifically, the faults of different types and defect diameters are seeded on the normal bearings by edM, including three single point faults and one combination fault, as shown in Figure 5. More detailed information about the bearing fault states in this experiment can be found in Table 1, in which the abbreviation of each state is defined for clarity. Furthermore, it should be noted that each experiment sample consists of 2000 data points. The time-domain waveforms of raw vibration signals for five states are presented in Figure 6. To validate the stable diagnosis performance of the proposed method, 10 repeated trials with the same setup are conducted in this case study. Notably, all experiments are implemented with MATLAB 2016 and the relevant program runs on a laptop with a CPU 3.2 GHz and 16 GB RAM.

### 3.2. Fault Diagnosis Results Analysis

#### 3.2.1. Multiscale Frequency Components Extraction by the SoVMD Algorithm

**Figure 7:**
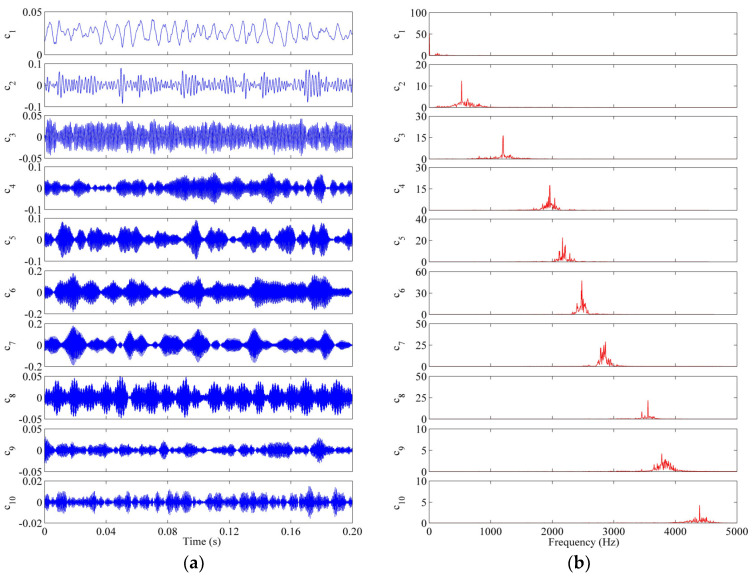
Waveforms of a fault sample of ‘RF_12′ state: (**a**) The obtained frequency components by SoVMD algorithm. (**b**) The corresponding frequency spectrum of components.

As the strong non-stationarity and nonlinearity of bearing vibration signals, the fault diagnosis accuracy would be obviously reduced if the process of feature extraction is directly executed using the entropy-based approach. In order to reduce the influence of signals complexity on diagnosis accuracy, as mentioned in Section 2.1, the collected signals should be decomposed first to learn the inherent characteristics of fault states from different frequency scales. Based on the developed SoVMD method, a series of multiscale frequency components $c_{i}\left(t\right)$ can be effectively obtained from raw signals. Specifically, the parameters related to the searching process are initialized as M=100, P=200, and $SS_{0}=100$. Furthermore, the searching intervals of K and α are set as $\left[3,15\right]$ and $\left[500,1500\right]$, respectively. Taking a signal sample of a roller fault with a 1.2 mm defect diameter (RF_12) as an example for analysis, Figure 7 lists the extracted frequency components of this sample by SoVMD and the corresponding frequency spectrum of these components. We can observe from this figure that the fault sample is decomposed into 10 frequency components and the spectra of these components are significantly different. The decomposition results mentioned above indicate that the problem of mode mixing can be effectively overcome using the SoVMD algorithm. In a follow-up study, based on the CSvDE theory, the obtained frequency components can be utilized to construct the MsFFS for bearing fault identification. 

#### 3.2.2. Analysis of CSvDE Scale Factor

**Figure 8:**
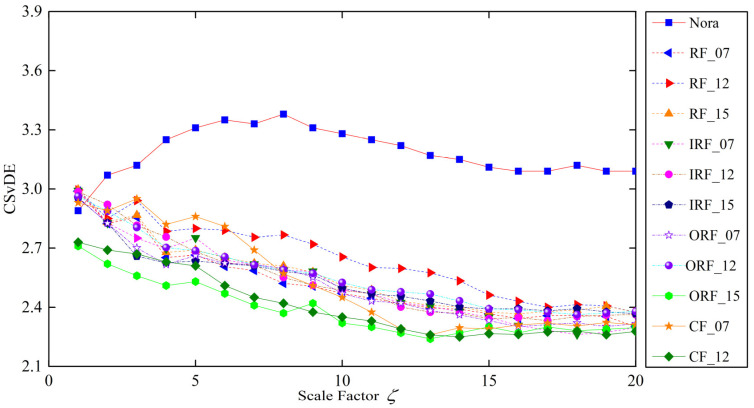
The development trend of CSvDE average values with the increase in scale factor.

As depicted in Equations (13) and (14), the maximum value of scale factor ζ should be determined preliminarily to construct the MsFFS of bearings for accurate fault diagnosis. For this, Figure 8 presents the development trend of CSvDE average values for all training samples under 12 operation states with the increase in scale factor, in which the scale factor varies from 1 to 20. More specifically, the remaining three parameters of CSvDE can be set as γ=4, m=6, and ξ=1 [35,36]. It can be found from the figure that the values of CSvDE show a gradual decreasing tendency with the scale factor increasing, regardless of the working state. Moreover, when ζ>15, the curve of CSvDE tends to be stable and there is obvious overlap between the CSvDE values of different fault states, which indicates that the current value of ζ is appropriate for fault identification. In other words, the interval of the scale factor for the construction of the MsFFS can be determined as $\zeta \in \left[1,15\right]$.

**Figure 9:**
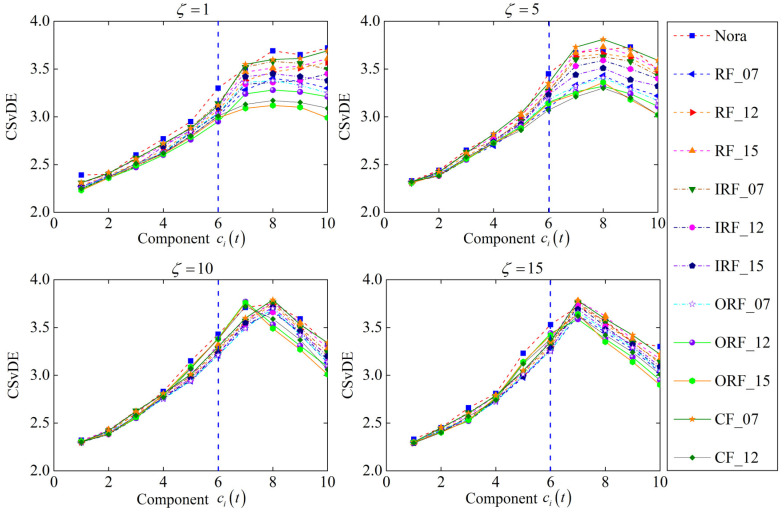
The CSvDE distributions of frequency components for 4 different scale factors.

In addition, to analyze the difference of CSvDE features of frequency components between 12 working states, the CSvDE values of all components under four different scale factors (ζ=1, 5, 10, 15) are calculated, as shown in Figure 9. It is obvious that the CSvDE values of the last few components are larger than those of the first several components when ζ=1, and a similar phenomenon occurs in the results of the other three factors. This is because the last few components with high frequency exhibit a stronger randomness and contain more information of fault states compared with the first several components. Moreover, it is worth noting that for the 12 states, the differences in CSvDE values of the last five components are more significant. However, there is obvious overlap between the CSvDE values of the other components. The relevant results indicate that the last five components show greater potential for identifying different operation states of bearings. Consequently, two groups of experiments can be conducted in this study, as described in Table 2. In other words, from the perspective of feature construction, we try to explore the influence of MsFFS structure on the diagnosis accuracy. And most remarkably, much more attention should be paid to Experiment 1 to demonstrate the superior performance of the proposed method.

Experiment 1: Without the process of frequency component selection, all components obtained by SoVMD are used to construct the MsFFS.

Experiment 2: Based on the analysis results mentioned above, the last five components are considered to construct the MsFFS. 

As depicted in Equation (21), the MsFFS can be effectively constructed for bearing fault diagnosis based on the extracted multiscale frequency components and CSvDE theory. Here, utilizing the results of the training set in Experiment 1 as an example, the following formula shows the constructed MsFFS to train the softmax classifier:


$$\left[\begin{array}{cccccc} Nora_{1}\left(CSvDE\left(c_{1},4,6,1,1\right)\right) & Nora_{1}\left(CSvDE\left(c_{1},4,6,1,2\right)\right) & \cdots & Nora_{1}\left(CSvDE\left(c_{1},4,6,1,15\right)\right) & \cdots & Nora_{1}\left(CSvDE\left(c_{10},4,6,1,15\right)\right)\\ \vdots & \vdots & \ddots & \vdots & \ddots & \vdots \\ Nora_{50}\left(CSvDE\left(c_{1},4,6,1,1\right)\right) & Nora_{50}\left(CSvDE\left(c_{1},4,6,1,2\right)\right) & \cdots & Nora_{50}\left(CSvDE\left(c_{1},4,6,1,15\right)\right) & \cdots & Nora_{50}\left(CSvDE\left(c_{10},4,6,1,15\right)\right)\\ RF_{-}07_{1}\left(CSvDE\left(c_{1},4,6,1,1\right)\right) & RF_{-}07_{1}\left(CSvDE\left(c_{1},4,6,1,2\right)\right) & \cdots & RF_{-}07_{1}\left(CSvDE\left(c_{1},4,6,1,15\right)\right) & \cdots & RF_{-}07_{1}\left(CSvDE\left(c_{10},4,6,1,15\right)\right)\\ \vdots & \vdots & \ddots & \vdots & \ddots & \vdots \\ RF_{-}07_{50}\left(CSvDE\left(c_{1},4,6,1,1\right)\right) & RF_{-}07_{50}\left(CSvDE\left(c_{1},4,6,1,2\right)\right) & \cdots & RF_{-}07_{50}\left(CSvDE\left(c_{1},4,6,1,15\right)\right) & \cdots & RF_{-}07_{50}\left(CSvDE\left(c_{10},4,6,1,15\right)\right)\\ \vdots & \vdots & \ddots & \vdots & \ddots & \vdots \\ CF_{-}12_{1}\left(CSvDE\left(c_{1},4,6,1,1\right)\right) & CF_{-}12_{1}\left(CSvDE\left(c_{1},4,6,1,2\right)\right) & \cdots & CF_{-}12_{1}\left(CSvDE\left(c_{1},4,6,1,15\right)\right) & \cdots & CF_{-}12_{1}\left(CSvDE\left(c_{10},4,6,1,15\right)\right)\\ \vdots & \vdots & \ddots & \vdots & \ddots & \vdots \\ CF_{-}12_{50}\left(CSvDE\left(c_{1},4,6,1,1\right)\right) & CF_{-}12_{50}\left(CSvDE\left(c_{1},4,6,1,2\right)\right) & \cdots & CF_{-}12_{50}\left(CSvDE\left(c_{1},4,6,1,15\right)\right) & \cdots & CF_{-}12_{50}\left(CSvDE\left(c_{10},4,6,1,15\right)\right) \end{array}\right]$$

In addition, to validate the more superior performance of the proposed method, six other approaches are also employed for rolling bearing fault diagnosis, including the EMD-CSvDE, VMD-CSvDE, SoVMD-multiscale sample entropy (MSE) [44], SoVMD-multiscale permutation entropy (MPE) [45], support vector machine (SVM) [46], and artificial neural network (ANN) [47]. More specifically, the first four comparison methods are designed to analyze the contribution of each link in the process of fault diagnosis, i.e., SoVMD-based multiscale frequency component extraction and CSvDE-based MsFFS construction. As described in Section 3.1, 10 trials with the same setup are implemented in two groups of experiments. Detailed descriptions about the parameter setup of seven methods in Experiment 1 are shown in Table 3.

**Figure 10:**
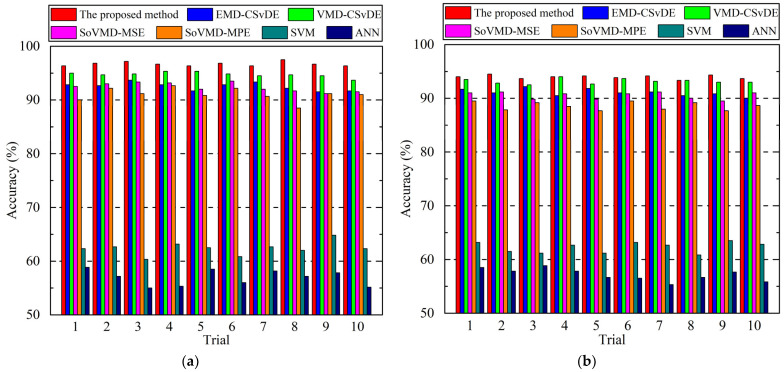
Detailed diagnosis accuracies of 10 trials for 7 methods. (**a**) Experiment 1. (**b**) Experiment 2.

**Figure 11:**
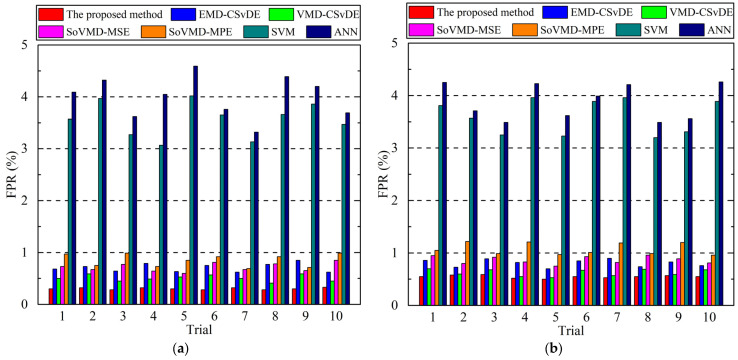
Detailed FPRs of 10 trials for 7 methods. (**a**) Experiment 1. (**b**) Experiment 2.

Considering the two different groups of experiments, the detailed diagnosis accuracies and FPRs of 10 trials for seven methods are presented in Figure 10 and Figure 11, respectively. Based on these results, the average accuracies and average false alarm rates of the seven methods in two experiments can be calculated and are listed in Table 4. In addition, to compare the implementation efficiency, the average computation time of 10 trials for different methods is shown in Table 5.

From the perspective of diagnosis accuracy and FPR of each trial, it can be seen from Figure 10 and Figure 11 that the accuracy and FPR of the proposed method are obviously superior to those of the other six approaches for both experiments. More comprehensively, as shown in Table 4, we can observe that the average accuracy and average FPR of the proposed method in Experiment 1 are 96.70% and 0.30%, respectively, which are slightly superior to EMD-CSvDE, VMD-CSvDE, SoVMD-MSE, and SoVMD-MPE, and significantly superior to SVM and ANN. Specifically, compared with the other six approaches, the accuracy of the proposed method in Experiment 1 is improved by 4.51%, 2.08%, 4.68%, 6.23%, 55.04%, and 69.89%, respectively, and the FPR is reduced by 55.88%, 41.18%, 57.14%, 63.41%, 91.60%, and 92.4%, respectively. Similar results also appear in Experiment 2 depicted in Table 4. Meanwhile, the standard deviations of the developed method for these two metrics are obviously smaller than those of the other approaches in any group of experiments, which confirms the stronger stability and robustness of the proposed method for bearing fault diagnosis. In addition, from the results presented in Table 5, it can be found that the average computation time of the proposed method is slightly more than those of EMD-CSvDE, VMD-CSvDE, SoVMD-MSE, and SoVMD-MPE, while it is much more than those of SVM and ANN, regardless of Experiment 1 or Experiment 2. Compared with the other four combined methods, the process of adaptive parameter optimization and MsFFS construction with variable scale factors in the framework of the proposed method will take more time to improve diagnosis accuracy. In addition, without the consideration of the strategies of signal decomposition and MsFFS construction, the computational costs of SVM and ANN will be reduced compared to those of the other five diagnosis methods.

**Figure 12:**
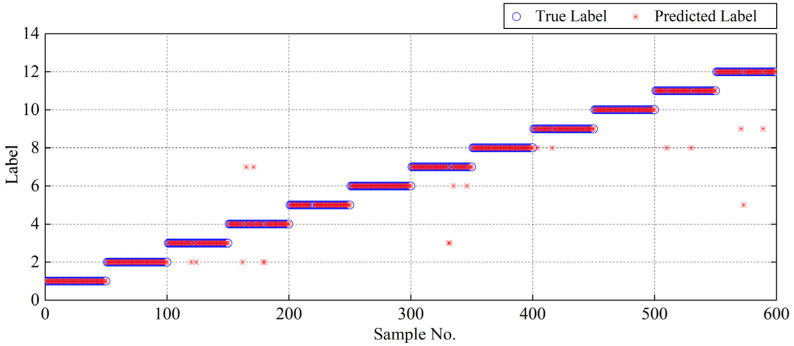
Detailed diagnosis results of the proposed method for the 6th trial in Experiment 1.

**Figure 13:**
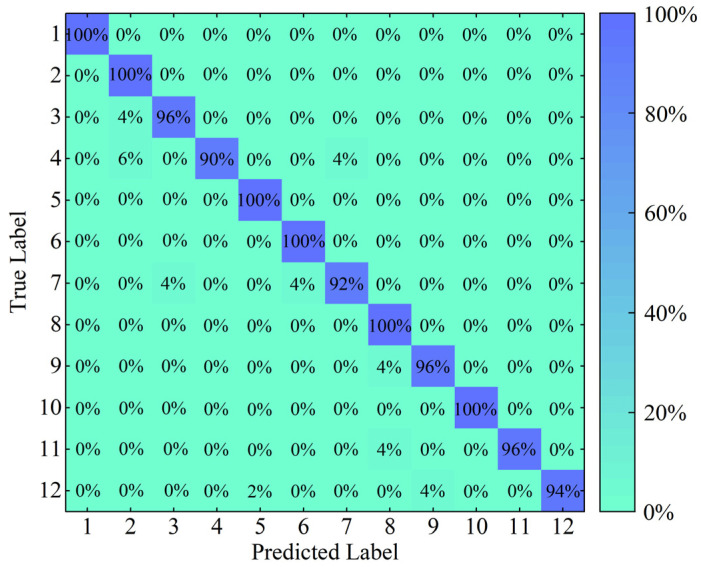
The multi-class confusion matrix of the proposed method for the 6th trial in Experiment 1.

To give more details, the fault diagnosis results of the proposed method for the sixth trial in Experiment 1 and the corresponding multi-class confusion matrix are shown in Figure 12 and Figure 13, respectively. In Figure 12, it can be seen clearly that a small number of predicted labels of testing samples deviate from the true labels, i.e., the phenomenon of misdiagnosis. More specifically, to intuitively reflect the accuracy rate and error rate, the multi-class confusion matrix can be further built based on the above-mentioned diagnosis results, as depicted in Figure 13. It can be observed from this figure that the diagnosis accuracy of different operation states can reach 90% or even higher, especially for six states (Nora, RF_07, IRF_07, IRF_12, ORF_07, and ORF_15) with an accuracy of 100%. Moreover, an overall accuracy of 97% can be achieved by the proposed method for the sixth trial in Experiment 1, which indicates that the proposed method contributes to identify the different fault types and defect severities of the rolling bearing and also realizes satisfactory diagnosis accuracy as a whole.

Through the above experiment results, the relevant conclusions can be summarized as follows. (1) Among all the diagnosis approaches, the proposed method realizes the highest diagnosis accuracy and the lowest FPR whether in Experiment 1 or Experiment 2, which strongly confirms its superior performance on bearing fault diagnosis. (2) Compared with the last two approaches (SVM and ANN), the remaining methods (the proposed method, EMD-CSvDE, VMD-CSvDE, SoVMD-MSE, and SoVMD-MPE) can accomplish the task of fault diagnosis with higher accuracy and lower FPR. The main reason is that the multiscale frequency component extraction by different decomposition algorithms contributes to capture the inherent characteristics of the raw signal and further establish an effective feature space for accurate diagnosis. (3) Adopting the SoVMD algorithm, the diagnosis accuracy and FPR of the developed method can be significantly improved compared with EMD-CSvDE and VMD-CSvDE. This is because the optimal parameters of the decomposition process can be adaptively determined by the SoVMD method so that the multiscale frequency components can be effectively obtained without the influence of the mode mixing problem. (4) From the perspective of feature space construction, the diagnosis performance of the proposed method is more excellent and stable than the other two approaches are, including SoVMD-MSE and SoVMD-MPE. Because of the variable parameters of scale factor, the developed CSvDE method is helpful to construct the MsFFS more effectively and improve the diagnosis performance compared with the methods of MSE and MPE. (5) For the same method, the diagnosis results in Experiment 1 are slightly superior to those in Experiment 2, but it is worth noting that the calculation time in Experiment 1 is obviously more than that in Experiment 2. This is because a small amount of fault information can still be contained in the first five frequency components and may be useful for accurate diagnosis. Taking the fewer components into account, the computation costs of Experiment 2 can be decreased significantly. 

**Figure 14:**
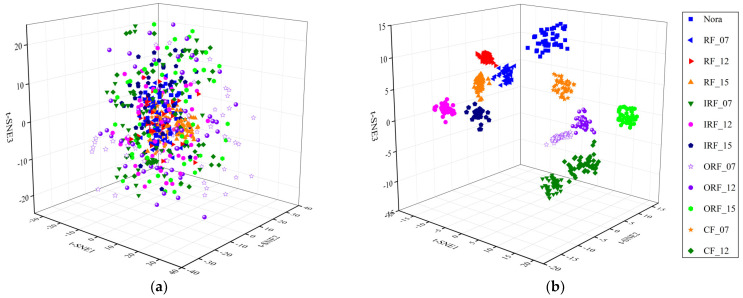
The three-dimensional projections by t-SNE in Experiment 1. (**a**) Original samples. (**b**) The MsFFS obtained by the proposed method.

Finally, to intuitively illustrate the superior performance on feature extraction of the proposed method, the extracted MsFFS can be reduced and visualized by the t-distributed stochastic neighbor embedding (t-SNE) algorithm. We use the results of the sixth trial in Experiment 1 as an example for analysis, and Figure 14 shows the three-dimensional projections of the original samples and the MsFFS by t-SNE. It is obvious that the MsFFS obtained by the proposed method can effectively reveal essential information contained in the original samples and accomplish the fault state identification with high accuracy. The main reason is that the complex nonlinear relationships between the raw signal and the MsFFS can be constructed effectively based on the model architecture integrating the component mode with a variable scale factor. To sum up, the proposed method can achieve superior performance in capturing valuable features for accurate fault diagnosis.

## 4. Conclusions

In this paper, a novel fault diagnosis method based on CSvDE and SoVMD, systematically integrating the nonstationary signal analysis method with machine learning technology, is proposed for rolling bearings of combine harvesters. Within the developed method, to solve the problems existing in traditional EMD and VMD approaches, a SoVMD algorithm is first designed to extract the multiscale frequency components from the raw signal sample. In essence, an adaptive parameter optimization method is presented in the theorical framework of SoVMD to conduct the search of VMD parameters with high efficiency. Compared with EMD, the mode mixing problem can be effectively tackled by the SoVMD method. Subsequently, an entropy-based feature construction theory, i.e., CSvDE, is presented to establish the MsFFS for fault diagnosis. Theoretically, the developed CSvDE fully blends the advantages of the variable scale of the parameter and DE. Compared with other entropies, such as MSE and MPE, the advantages of CSvDE result in more accurate and stable results. The results of a case study of the rolling bearing datasets of combine harvesters show that the proposed method has a more excellent and robust diagnosis performance than other existing approaches. Nevertheless, the computation consumption is relatively high; thus, decreasing the time cost and, at the same time, guaranteeing satisfactory accuracy are still valuable topics to be further explored in the future. 

## Figures and Tables

**Table 1:** Descriptions of rolling bearing fault states in the experiments.

States		Abbreviation	Number of Samples		Label
Fault Type	Defect Diameter (mm)		Training Set	Testing Set	
Normal		Nora	50	50	1
Roller fault	0.7	RF_07	50	50	2
	1.2	RF_12	50	50	3
	1.5	RF_15	50	50	4
Inner ring fault	0.7	IRF_07	50	50	5
	1.2	IRF_12	50	50	6
	1.5	IRF_15	50	50	7
Outer ring fault	0.7	ORF_07	50	50	8
	1.2	ORF_12	50	50	9
	1.5	ORF_15	50	50	10
Combination fault	0.7	CF_07	50	50	11
	1.2	CF_12	50	50	12

**Table 2:** The relevant information about the two groups of experiments.

Experiment Group	Description	The Dimension of MsFFS	
		Training Set	Testing Set
1	All components should be used to construct the MsFFS	600 × 150	600 × 150
2	The last five components are adopted to construct the MsFFS	600 × 75	600 × 75

**Table 3:** Descriptions about the parameter setup of seven methods in Experiment 1.

Methods	Parameter Setup
The proposed method	For SoVMD, the maximum number of iterations is 1000, and the number of components and the penalty factor are optimized between [3, 15] and [500, 1500], respectively. For CSvDE, the embedding dimension, category number, and time delay are set as 4, 6, and 1, respectively, and the scale factor varies between 1 and 15.
EMD-CSvDE	For EMD, the maximum number of iterations is 1000. For CSvDE, the corresponding parameters are set as the same as that of the proposed method.
VMD-CSvDE	For VMD, the maximum number of iterations is 1000, and the number of components and the penalty factor are arbitrarily determined as 7 and 1000, respectively. For CSvDE, the corresponding parameters are set as the same as that of the proposed method.
SoVMD-MSE	For SoVMD, the corresponding parameters are set as the same as that of the proposed method. For MSE, the embedding dimension and time delay are set as 4 and 1, respectively, and the scale factor varies between 1 and 15.
SoVMD-MPE	For SoVMD, the corresponding parameters are set as the same as that of the proposed method. For MPE, the embedding dimension and time delay are set as 4 and 1, respectively, and the scale factor varies between 1 and 15.
SVM	The RBF is used as the kernel function. The penalty factor is set as 3, and the kernel radius is set as 1.
ANN	The structure of the network is 2000-300-12. The learning rate and momentum are 0.1 and 0.3, respectively, and the maximum number of iterations is 1000.

**Table 4:** Comparison of average accuracies and average FPRs of 7 methods in two experiments.

Methods	Experiment 1		Experiment 2	
	Accuracy (%)	FPR (%)	Accuracy (%)	FPR (%)
The proposed method	96.70 ± 0.38	0.30 ± 0.02	93.97 ± 0.33	0.55 ± 0.03
EMD-CSvDE	92.52 ± 0.68	0.68 ± 0.08	91.07 ± 0.61	0.81 ± 0.09
VMD-CSvDE	94.73 ± 0.46	0.51 ± 0.06	93.17 ± 0.43	0.62 ± 0.07
SoVMD-MSE	92.38 ± 0.69	0.70 ± 0.09	90.52 ± 0.61	0.86 ± 0.09
SoVMD-MPE	91.03 ± 0.81	0.82 ± 0.11	88.57 ± 0.70	1.05 ± 0.13
SVM	62.37 ± 1.16	3.57 ± 0.34	62.28 ± 1.19	3.61 ± 0.33
ANN	56.92 ± 1.37	3.99 ± 0.40	57.15 ± 1.26	3.92 ± 0.38

**Table 5:** Comparison of average computation time of 7 methods in two experiments.

Methods	Average Computation Time in Experiment 1 (s)		Average Computation Time in Experiment 2 (s)	
	For Training Set	For Testing Set	For Training Set	For Testing Set
The proposed method	87.15	69.21	62.34	51.05
EMD-CSvDE	79.79	58.03	52.05	43.91
VMD-CSvDE	71.53	52.82	47.97	40.67
SoVMD-MSE	86.06	66.52	57.16	48.62
SoVMD-MPE	82.23	64.71	55.62	45.39
SVM	22.07	15.55	21.53	15.67
ANN	27.92	23.99	26.86	23.03

## Data Availability

Data is contained within the article.

## Nomenclature

CSvDE	Composite-Scale-variable Dispersion Entropy
SoVMD	Self-optimization Variational Mode Decomposition
MsFFS	Multiscale Fault Features Space
WT	Wavelet Transform
EMD	Empirical Mode Decomposition
EWT	Empirical Wavelet Transform
EEMD	Ensemble Empirical Mode Decomposition
VMD	Variational Mode Decomposition
AE	Approximate Entropy
SE	Sample Entropy
PE	Permutation Entropy
DE	Dispersion Entropy
MSE	Multiscale Sample Entropy
MPE	Multiscale Permutation Entropy
SVM	Support Vector Machine
ANN	Artificial Neural Network
FPR	False Alarm Rate
t-SNE	T-distributed stochastic neighbor embedding

## References

[1] Qiu Z., Shi G., Zhao B., Jin X., Zhou L. (2022). Combine harvester remote monitoring system based on multi-source information fusion. Comput. Electron. Agr..

[2] Yang G., Cheng Y., Xi C., Liu L., Gan X. (2022). Combine Harvester Bearing Fault-Diagnosis Method Based on SDAE-RCmvMSE. Entropy.

[3] Ma R., Wang X., Ni Z., Zeng C. (2022). Dual-impulse behavior analysis and quantitative diagnosis of the raceway fault of rolling bearing. Mech. Syst. Signal Process..

[4] Yan G., Chen J., Bai Y., Yu C., Yu C. (2022). A Survey on Fault Diagnosis Approaches for Rolling Bearings of Railway Vehicles. Processes.

[5] Wang J., Liu P., Lu S., Zhou M., Chen X. (2023). Decentralized plant-wide monitoring based on mutual information-Louvain decomposition and support vector data description diagnosis. ISA Trans..

[6] Wang J., Lv X., Meng Z., Puig V. (2023). An integrated design method for active fault diagnosis and control. Int. J. Robust Nonlinear Control..

[7] Ahmed H., Nandi A. (2022). Intrinsic Dimension Estimation-Based Feature Selection and Multinomial Logistic Regression for Classification of Bearing Faults Using Compressively Sampled Vibration Signals. Entropy.

[8] Wang H., Chen K., Lin L. (2021). Bearing fault diagnosis based on the active energy conversion of generalized stochastic resonance in fluctuating-frequency linear oscillator. Meas. Sci. Technol..

[9] Liu Q., Wang Y., Xu Y. (2021). Synchrosqueezing extracting transform and its application in bearing fault diagnosis under non-stationary conditions. Measurement.

[10] Liang P., Wang W., Yuan X., Liu S., Zhang L., Cheng Y. (2022). Intelligent fault diagnosis of rolling bearing based on wavelet transform and improved ResNet under noisy labels and environment. Eng. Appl. Artif. Intell..

[11] Liu Q., Yang J., Zhang K. (2022). An improved empirical wavelet transform and sensitive components selecting method for bearing fault. Measurement.

[12] Ye X., Hu Y., Shen J., Chen C., Zhai G. (2021). An Adaptive Optimized TVF-EMD Based on a Sparsity-Impact Measure Index for Bearing Incipient Fault Diagnosis. IEEE Trans. Instrum. Meas..

[13] Zhong T., Qu J., Fang X., Li H., Wang Z. (2021). The intermittent fault diagnosis of analog circuits based on EEMD-DBN. Neurocomputing.

[14] Dragomiretskiy K., Zosso D. (2014). Variational Mode Decomposition. IEEE Trans. Signal Process..

[15] Zhou J., Xiao M., Niu Y., Ji G. (2022). Rolling Bearing Fault Diagnosis Based on WGWOA-VMD-SVM. Sensors.

[16] Li C., Liu Y., Liao Y., Wang J. (2022). A VME method based on the convergent tendency of VMD and its application in multi-fault diagnosis of rolling bearings. Measurement.

[17] Jin Z., Chen G., Yang Z. (2022). Rolling Bearing Fault Diagnosis Based on WOA-VMD-MPE and MPSO-LSSVM. Entropy.

[18] Wang Q., Yang C., Wan H., Deng D., Nandi A. (2022). Bearing fault diagnosis based on optimized variational mode decomposition and 1D convolutional neural networks. Meas. Sci. Technol..

[19] Zhao D., Li J., Cheng W., Wen W. (2023). Bearing multi-fault diagnosis with iterative generalized demodulation guided by enhanced rotational frequency matching under time-varying speed conditions. ISA Trans..

[20] Wang J., Zhang W., Wu H., Zhou J. (2021). Improved bilayer convolution transfer learning neural network for industrial fault detection. Can. J. Chem. Eng..

[21] Zhao D., Cui L., Liu D. (2022). Bearing Weak Fault Feature Extraction Under Time-Varying Speed Conditions Based on Frequency Matching Demodulation Transform. IEEE/ASME Trans. Mechatron..

[22] Zhao D., Wang T., Chu F. (2019). Deep convolutional neural network based planet bearing fault classification. Comput. Ind..

[23] Wang X., Si S., Li Y. (2022). Variational Embedding Multiscale Diversity Entropy for Fault Diagnosis of Large-Scale Machinery. IEEE Trans. Ind. Electron..

[24] Li Y., Gao P., Tang B., Yi Y., Zhang J. (2022). Double Feature Extraction Method of Ship-Radiated Noise Signal Based on Slope Entropy and Permutation Entropy. Entropy.

[25] Huo Z., Martinez-Garcia M., Zhang Y., Yan R., Shu L. (2020). Entropy Measures in Machine Fault Diagnosis: Insights and Applications. IEEE Trans. Instrum. Meas..

[26] Wang X., Si S., Li Y. (2022). Hierarchical diversity entropy for the early fault diagnosis of rolling bearing. Nonlinear Dynam..

[27] Pincus S. (1991). Approximate entropy as a measure of system complexity. Proc. Nat. Acad. Sci. USA.

[28] Zhao S., Liang L., Xu G., Wang J., Zhang W. (2013). Quantitative diagnosis of a spall-like fault of a rolling element bearing by empirical mode decomposition and the approximate entropy method. Mech. Syst. Signal Process..

[29] Liu W., Jiang Y., Xu Y. (2022). A Super Fast Algorithm for Estimating Sample Entropy. Entropy.

[30] Jiang W., Zhou J., Xu Y., Liu J., Shan Y. (2019). Multistep Degradation Tendency Prediction for Aircraft Engines Based on CEEMDAN Permutation Entropy and Improved Grey-Markov Model. Complexity.

[31] Gao Y., Karimi M., Kudreyko A., Song W. (2018). Spare optimistic based on improved ADMM and the minimum entropy de-convolution for the early weak fault diagnosis of bearings in marine systems. ISA Trans..

[32] Tian Y., Wang Z., Lu C. (2019). Self-adaptive bearing fault diagnosis based on permutation entropy and manifold-based dynamic time warping. Mech. Syst. Signal Process..

[33] Richman J., Moorman J. (2000). Physiological time-series analysis using approximate entropy and sample entropy. Am. J. Physiol.-Heart Circ. Physiol..

[34] Huo Z., Zhang Y., Jombo G., Shu L. (2020). Adaptive Multiscale Weighted Permutation Entropy for Rolling Bearing Fault Diagnosis. IEEE Access.

[35] Rostaghi M., Azami H. (2016). Dispersion Entropy: A Measure for Time-Series Analysis. IEEE Signal Process. Lett..

[36] Rostaghi M., Ashory M., Azami H. (2018). Application of dispersion entropy to status characterization of rotary machines. J. Sound Vib..

[37] Wang Z., Yang N., Li N., Du W., Wang J. (2021). A new fault diagnosis method based on adaptive spectrum mode extraction. Struct. Health.

[38] Shao K., He Y., Liu X., Xing Z., Du B. (2022). Fault Detection for Wind Turbine System Using Fractional Extended Dispersion Entropy and Cumulative Sum Control Chart. IEEE Trans. Instrum. Meas..

[39] Zhao D., Liu S., Cheng S., Sun X., Wang L., Wei Y., Zhang H. (2021). Parallel multi-scale entropy and it’s application in rolling bearing fault diagnosis. Measurement.

[40] Pan Z., Cai F., Chen W., Chen H. (2022). Graph Co-Attentive Session-based Recommendation. ACM Trans. Inf. Syst..

[41] Cheng C., Zhou B., Ma G., Wu D., Yuan Y. (2020). Wasserstein distance based deep adversarial transfer learning for intelligent fault diagnosis with unlabeled or insufficient labeled data. Neurocomputing.

[42] Maldonado J., Riff M., Neveu B. (2022). A review of recent approaches on wrapper feature selection for intrusion detection. Expert Syst. Appl..

[43] Lv M., Liu S., Chen C. (2022). A New Feature Extraction Technique for Early Degeneration Detection of Rolling Bearings. IEEE Access.

[44] Jiao W., Li G., Jiang Y., Baim R., Tang C., Yan T., Ding X., Yan Y. (2021). Multi-Scale Sample Entropy-Based Energy Moment Features Applied to Fault Classification. IEEE Access.

[45] Zhao D., Liu S., Gu D., Sun X., Wang L., Wei Y., Zhang H. (2020). Improved multi-scale entropy and it’s application in rolling bearing fault feature extraction. Measurement.

[46] Song R., Yu B., Shi H., Yang L., Dong Z. (2023). Support vector machine fault diagnosis based on sparse scaling convex hull. Meas. Sci. Technol..

[47] Yang Z., Kong C., Wang Y., Rong X., Wei L. (2021). Fault diagnosis of mine asynchronous motor based on MEEMD energy entropy and ANN. Comput. Electr. Eng..

